# A Numerical Approach to Ion Channel Modelling Using Whole-Cell Voltage-Clamp Recordings and a Genetic Algorithm

**DOI:** 10.1371/journal.pcbi.0030169

**Published:** 2007-08-31

**Authors:** Meron Gurkiewicz, Alon Korngreen

**Affiliations:** 1 The Mina and Everard Goodman Faculty of Life Sciences, Bar-Ilan University, Ramat-Gan, Israel; 2 The Leslie and Susan Gonda Multidisciplinary Brain Research Center, Bar-Ilan University, Ramat-Gan, Israel; UFR Biomédicale de l'Université René Descartes, France

## Abstract

The activity of trans-membrane proteins such as ion channels is the essence of neuronal transmission. The currently most accurate method for determining ion channel kinetic mechanisms is single-channel recording and analysis. Yet, the limitations and complexities in interpreting single-channel recordings discourage many physiologists from using them. Here we show that a genetic search algorithm in combination with a gradient descent algorithm can be used to fit whole-cell voltage-clamp data to kinetic models with a high degree of accuracy. Previously, ion channel stimulation traces were analyzed one at a time, the results of these analyses being combined to produce a picture of channel kinetics. Here the entire set of traces from all stimulation protocols are analysed simultaneously. The algorithm was initially tested on simulated current traces produced by several Hodgkin-Huxley–like and Markov chain models of voltage-gated potassium and sodium channels. Currents were also produced by simulating levels of noise expected from actual patch recordings. Finally, the algorithm was used for finding the kinetic parameters of several voltage-gated sodium and potassium channels models by matching its results to data recorded from layer 5 pyramidal neurons of the rat cortex in the nucleated outside-out patch configuration. The minimization scheme gives electrophysiologists a tool for reproducing and simulating voltage-gated ion channel kinetics at the cellular level.

## Author Summary

Voltage-gated ion channels affect neuronal integration of information. Some neurons express more than ten different types of voltage-gated ion channels, making information processing a highly convoluted process. Kinetic modelling of ion channels is an important method for unravelling the role of each channel type in neuronal function. However, the most commonly used analysis techniques suffer from shortcomings that limit the ability of researchers to rapidly produce physiologically relevant models of voltage-gated ion channels and of neuronal physiology. We show that conjugating a stochastic search algorithm with ionic currents measured using multiple voltage-clamp protocols enables the semi-automatic production of models of voltage-gated ion channels. Once fully automated, this approach may be used for high throughput analysis of voltage-gated currents. This in turn will greatly shorten the time required for building models of neuronal physiology to facilitate our understanding of neuronal behaviour.

## Introduction

Ion channels are trans-membrane proteins that close and open in reaction to changes in membrane potential, among other factors, thus leading to a change in ion flow across the membrane. Membrane potential may be the most significant factor affecting the activity of ion channels, for not only do the kinetics of many channels depend on membrane potential but also changes in membrane potential are the main instigator of neuronal activity [1,2]. The kinetics of these voltage-dependent ion channels are complex, requiring the construction of intricate kinetic models to understand ion channel behaviour.

The dominant paradigm for ion transport over the past 50 years is based on the seminal experiments of Hodgkin and Huxley [3–8]. Their detailed kinetic models derived from the giant axon of *Loligo* are still extremely useful in studies of ion channels and of neuronal physiology. However, a much more detailed picture of the mechanisms underlying membrane excitation has emerged over the years, emphasizing several disagreements with the Hodgkin-Huxley model. These include their proposed lack of connectivity between the activating and inactivating “gates” of the voltage-dependent sodium channel [9] and their premise that the inactivation gate voltage dependence is due to its coupling to the activation process; i.e., the inactivation gate can close only after the activation gate opens [10,11]. Nevertheless, the Hodgkin-Huxley model is still predominant in many simulations of neuronal physiology, mostly due to its ease of use and conceptualization combined with the relative small number of free parameters needing estimation for the quantification of channel behavior.

Most models proposed to replace the Hodgkin-Huxley model still use the same kinetic formalism and are only satisfactory in explaining certain aspects of channel behaviour but fail in others. For example, the classical Hodgkin-Huxley paradigm does not consider interactions between varying kinetic states, particularly that between activation and inactivation. This failure leads to an erroneous estimation of the kinetic parameters and thus of the predicted channel dynamics [12]. The best method, so far, for discovering the kinetics of ionic channels is by analysis of single-channel recordings [13]. However, single-channel recording and analysis suffer from several problems. One needs to accurately subtract the capacity of the electrodes, and the analysis of first latencies is extremely difficult [13–18]. Most data on neuronal voltage-gated conductances obtained in studies of cellular physiology have thus been collected when many channels were activated simultaneously, either in the whole-cell mode or in excised patches containing many channels. That is, most models of voltage-gated channels that aim to explain the physiological function of the channels are generated by analysing simultaneous activity in many channels.

Here we propose a method for analyzing whole-cell recordings of voltage-gated channels. Our working hypothesis is that it is possible to verify the viability of voltage-dependent ion channel models using a genetic optimization algorithm concurrently with a full-trace fit of experimental data to the model. We therefore scanned several of the better-known models of voltage-gated sodium and potassium channels to examine their accuracy in predicting and reproducing measured currents. Though the data provided for this paper derive from somata of L5 pyramidal neurons of the rat cortex, the method suggested here is applicable to all types of voltage-clamp recordings from different neuron classes. Note that our data were obtained using the nucleated patch configuration; thus, the models proposed here are not as detailed as those that may be obtained using single-channel recording. However, they are functionally significant models which allow us (and hopefully future researchers) to predict, simulate, and analyze neuronal physiology.

## Methods

### Simulation Environment

The model and the genetic algorithm (GA) were programmed using NEURON 5.7 and 5.8 [19]. We parallelized the process using a cluster of ten Pentium 4 computers with a 3 GHz clock speed sharing the same network file system (NFS). One of the machines functioned as a master, submitting and managing the jobs using a Parallel Virtual Machine (PVM), while the rest were slaves, reading and writing information from a shared directory in the network file system. Ion channel models were implemented using the NMODL extension of NEURON [20]. Results were analyzed using custom procedures written in IgorPro 5.01 (Wavemetrics, http://www.wavemetrics.com/).

### Genetic Algorithm

A GA is a search algorithm based on the mechanisms of Darwinian evolution. It uses random mutation, crossover, and selection operators to breed better models or solutions (individuals) from an originally random starting population [21]. In this study, we started each search with a random population that was at least 20 times larger than the number of free parameters in the fitted model. Each individual in the population described a parameter set and the model was evaluated for each one of them. A search space was defined for each parameter; this avoided parameter combinations causing instability to the set of differential equations, while covering most of the physiological range expected for the parameters. Thus, for rate constants (k) the range was set from zero to 2,000 s^−1^, for voltage-dependence parameters (z) from zero to 2000 V^−1^, and zero to 100 pS for the conductance. Only in model C, where a parameter describing a voltage shift was required, was the range set from zero to +100 mV, disregarding the negative range of this parameter, since the expected value for this parameter was positive in both the simulated and the real data.

The population was sorted according to the value of the cost function (Equation 1) of each individual, and a new generation was created using selection, crossover, and mutation as operators. Selection used a tournament in which two pairs of individuals were randomly selected and the individual with the better score from each pair was transferred to the next generation. This procedure was repeated N/2 times (where N is the size of the population) until the new population was full. The one exception to this selection process (and later to the crossover and mutation operators) was the best individual that was transferred unchanged to the next generation to prevent a genetic drift.

**Figure 1:**
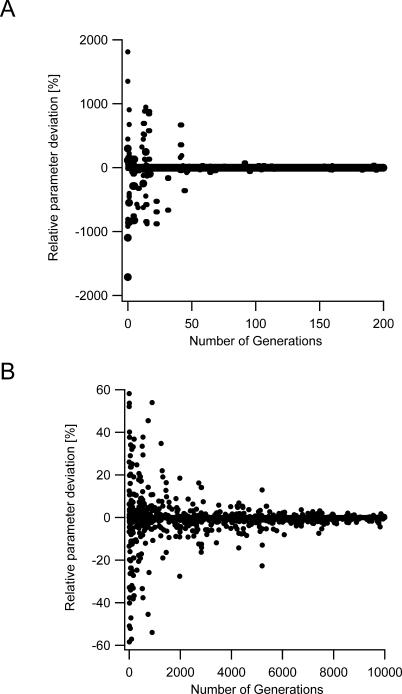
Progression of the Best Individual (A) The relative changes in the values of the parameters in the best individual were calculated by the following equation *F* = 100 · (*P_j_*_,*N*_ − *P_j_*_,*N*−1_)/*P_j_*_,N−1_ where *P_j_*_,N_ denotes the value of the *j* ^th^ parameter of the best individual in generation *N* and *P_j_*_,N−1_ denotes the value of the same parameter in the previous generation. The values were multiplied by 100 to display percentage changes. The graph contains the analysis of all parameters in a nine-parameter model without differentiating between them. (B) Same graph as in (A) with a longer generation scale and a zoom in on the parameter deviation scale.

**Figure 2:**
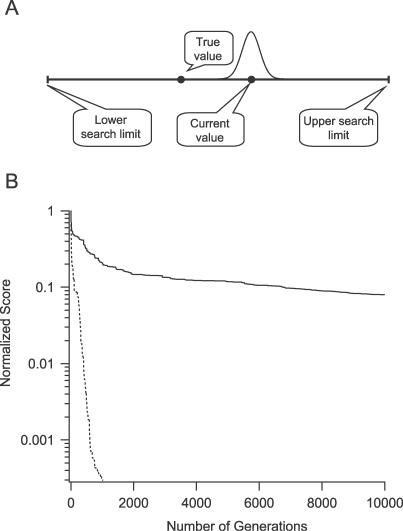
Modification of the Genetic Algorithm (A) A schematic drawing of the changes made to the GA as described in the text. (B) Convergence of the GA when the algorithm searched the entire search domain (smooth line) and when the search domain was focused around the current best values 200 generations after the start of the search (dashed line).

Each pair selected for transfer to the new population was subjected to a one-point crossover operator with a probability of 0.5. After the new population was created, each parameter value in the new population was subjected to mutation with a probability of 0.01. This allowed, albeit with a low occurrence frequency, the creation of double and even triple mutations to the same individual, thus increasing the variability in the new population. As detailed in Figures 1 and 2, two types of mutation operators were used. The first was a substitution of the parameter value with a random value drawn from a flat random number distribution that spanned the entire search space of the parameter. The second mutation operator, depicted in Figure 2, was a relative operator which changed the value of a parameter relative to its current value using a random number drawn from a Gaussian distribution centred on the current value of the parameter with a relative variance of 5%. We tested several values of mutation and crossover probabilities and found these values to be the optimal for the current project.

Ideally, the termination criterion should be that some cost function reaches a value of zero. In practice this is not possible since the run time of the process is limited and reaching this score can take a long time. The simulations ranged from less than an hour for a simple model with a small amount of data (see also the demonstration code in  Text S1, which takes two hours on a single Pentium 4 with a 3 GHz clock speed and less than 20 minutes on our cluster and the animation of the convergence of the GA to a model of a voltage-gated ion channel in Video S1) to more than a week for a 20-parameter model fitting many experimental points. Therefore, the process was terminated when the value of the best individual had not changed for several hundred generations. Depending on the complexity of the model, this occurred after 1,000–30,000 generations.

The cost function calculated root mean distance between the target and the test ionic current:

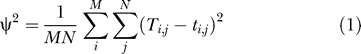
where *T* represents the target data set and *t* the test dataset. *N* was the total number of points in each simulated ionic current trace and *M* the number of voltage-clamp sweeps simulated in the model.

To rank the ability of various models to fit the data, we used the Log Error Ratio (LER):

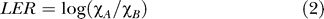
where *χ_A_* and *χ_B_* are the sum of squared errors for fitting the data to models A and B, respectively [22]. Equation 2 applies in theory to models containing a similar number of parameters. This can be corrected for by using the asymptotic information criterion *AIC* = 2 · (*NP_A_* − *NP_B_*) / *n* [23], where *NP_A_* and *NP_B_* are the number of free parameters in each model and *n* is the number of data points. In this study a large dataset with 15,000–30,000 data points was used for fitting. Therefore, the *AIC* correction was small and not applied in the calculations. Only LER values are reported.

### Channel Models

The models used to generate simulated currents are described below. Some minor changes to these models were made when voltage-gated K^+^ currents recorded from nucleated patches were analyzed. The modifications are noted in Table 1. Moreover, many published models contain mathematical expressions that hinder the proper use of minimization algorithms. For example, a common expression of a rate constant in a model for a voltage-gated channel can often be seen to assume the general form, *k* = *A*exp(−*z*(*V* − *V*_1/2_)). However, the expression exp(*zV*_1/2_) can also be expressed as part of the pre-exponential value leading to a simpler equation *k* = *A′*exp(−*zV*). Using the former expression in a minimization scheme invariably leads the algorithm astray due to the interchangeability of the pre-exponential and the fixed parameters in the exponent. Therefore, in all the models we have taken from the modeling literature and that appear below we verified that such interchangeability was eliminated by modifying the formal description of the model.

**Table 1:**
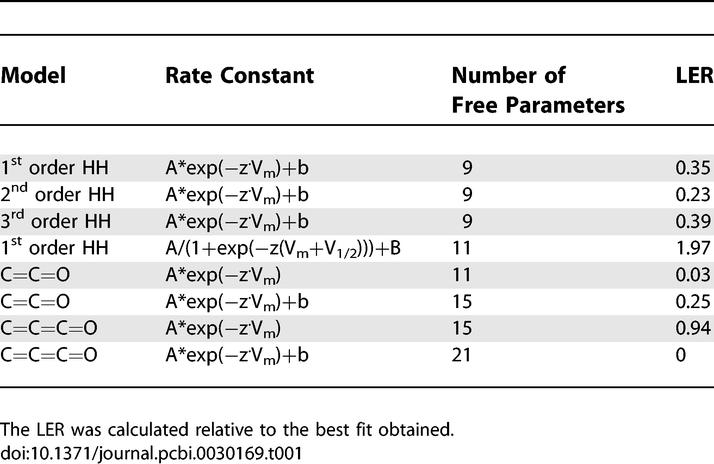
Models Tested in the Fit of the K^+^ Currents Displayed in Figure 10

#### Model A.

A nine-parameter potassium channel Markov model consisting of two closed states and one open state. Rate constants were defined as

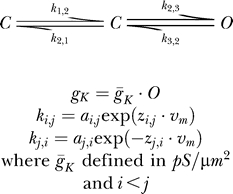



**Figure 3:**
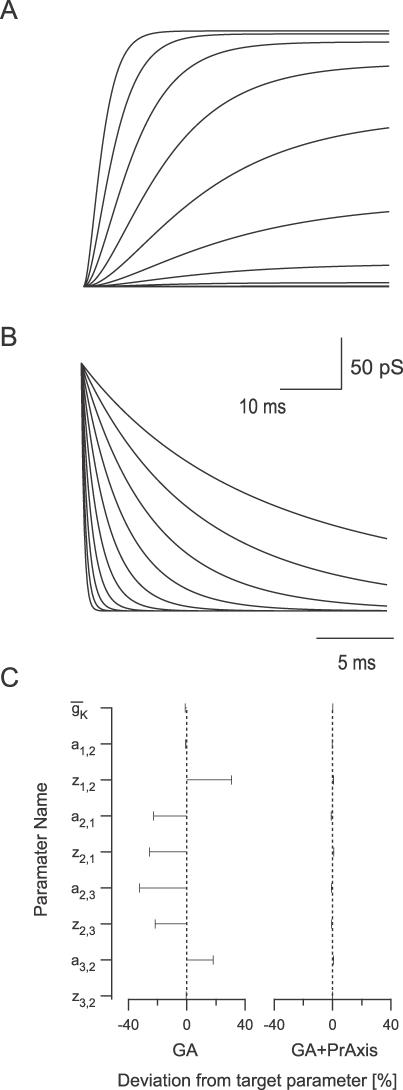
Constraining a Nine-Parameter Voltage-Gated K^+^ Channel Model (A) Activation of the conductance of Model A (see Methods) simulated in response to depolarizing voltage steps from −40 to +60 mV in steps of 20 mV. (B) Deactivation of the conductance of Model A. Following a 20-ms activation to +60 mV, deactivation was simulated by lowering potential to −120 mV to −20 mV in steps of 10 mV. (C) The errors in the best parameter set obtained by the GA (left) and GA followed by PrAxis (right) are plotted relative to their target values (dotted lines).

The values of each component of the rate constants for this model were 0.05, while the maximal conductance value was 20 (Figure 3).

#### Model B.

A 13-parameter potassium channel Markov model consisting of three closed states and one open state. Rate constants were defined as

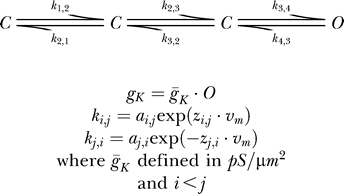



**Figure 4:**
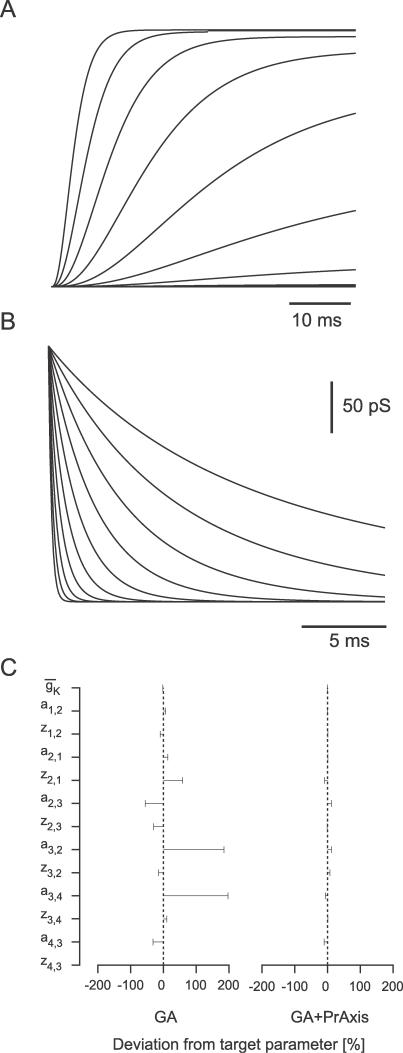
Constraining a 13-Parameter Voltage-Gated K^+^ Channel Model (A) Activation of the conductance of Model B (see Methods) simulated in response to depolarizing voltage steps from −40 to +60 mV in steps of 20 mV. (B) Deactivation of the conductance of Model A. Following a 20 ms activation to +60 mV, deactivation to potentials ranging from −120 to −20 mV in steps of 10 mV was simulated. (C) The errors in the best parameter set obtained by the GA (left) and GA followed by PrAxis (right) plotted relative to their target values (dotted lines).

The values of each component of the rate constants for this model were 0.05, while the maximal conductance value was 20 (Figure 4).

#### Model C.

**Figure 5:**
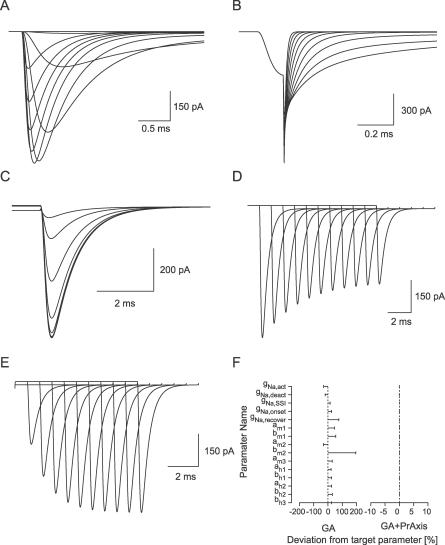
Constraining a 15-Parameter Model of a Voltage-Gated Na^+^ Channel (A) Simulated activation of a Hodgkin-Huxley–like model of the voltage-gated Na^+^ current (Model C, [24]) in response to depolarizing voltage commands. (B) Simulated deactivation protocol. (C) Simulated steady-state inactivation. (D) Simulated pulse inactivation. (E) Simulated recovery from inactivation. (F) The errors in the best parameter set obtained by the GA (left) and GA followed by PrAxis (right) plotted relative to their target values (dotted lines).

A 16-parameter Hodgkin-Huxley–like model based on the retinal ganglion cell voltage–dependent sodium channel model [24]. Some changes were made to the original model due to mathematical redundancies which, as detailed above, may lead to an infinite number of variable combinations producing correct fits. We therefore changed the model as follows for our simulated experiments (Figure 5):

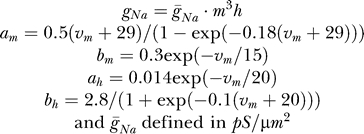



This produces a model with ten variables instead of twelve. Five additional parameters were the maximal conductances for each of the simulated voltage stimulation protocols, which were given the same value as in Models A and B in the simulated experiments. The reversal potential for sodium ions was defined as the sixteenth variable when the model was fit to experimental data, producing the equations:

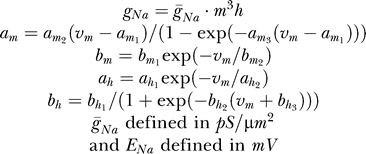



#### Model D.

**Figure 6:**
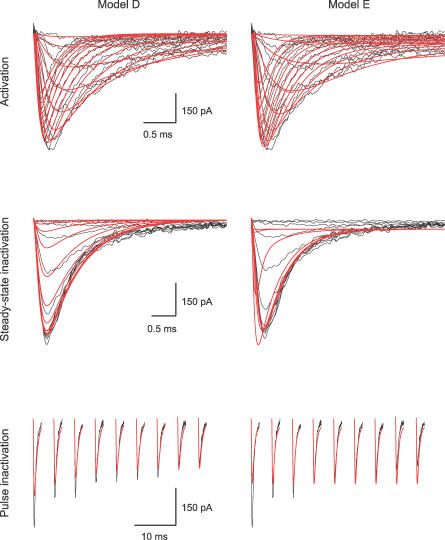
Fitting a Model to Experimentally Recorded Voltage-Gated Na^+^ Currents from Neocortical Pyramidal Neurons Results of fitting a dataset containing three pulse protocols to two models, Model D [25], left column, and Model E [16], right column. Data are shown as black lines, the fit in red. Pre-pulses and long stretches of data recorded at the holding potential were removed from the data traces to reduce possible bias to the fit. The activation traces (top traces) are inward currents recorded from a nucleated patch. A 100-ms pulse to −110 mV followed by a 20-ms pulse to voltages between −30 and +35 mV at 5-mV increments. Steady-state inactivation is shown in the middle traces. The inward currents were generated by a 20-ms voltage step to zero following a 100-ms pre-pulse to voltages between −105 mV and −5 mV at 10-mV increments. In pulse inactivation (bottom traces), the inward currents were generated in response to a voltage step to −50 mV (following a 100-ms pre-pulse to −110 mV) for varying durations from 0 ms to 45 ms in increments of 5 ms, followed by another voltage step to 0 ms for 30 ms. Data in all traces were filtered at 10 kHz and sampled at 20 kHz. The parameters generating the fit of Model D were: g_Na,act_ = 20.65; g_Na,SSI_ = 20.44; g_Na,onset_ = 12.15; a_1,2_ = 12.27; z_1,2_ = 0.038; a_2,1_ = 0.01; z_2,1_ = 0.246; a_2,3a_ = 21.55; a_2,3b_ = 1.42; z_2,3_ = 0.130; a_3,2a_ = 1428; a_3,2b_ = 234.5; z_3,2_ = 0.0047; a_1,4a_ = 3.9; a_1,4b_ = 1.72; z_1,4_ = 0.08; a_4,1_ = 0.015; z_4,1_ = 0.031; and E_Na_=59.7 mV.

A 20-parameter model based on the voltage-dependent sodium channel model [25]. Several modifications were made to this model to rid it of mathematical redundancies. First, redundancies such as those mentioned in Model C were eliminated. The next step was to make sure, through basic arithmetic, that in each Boltzmann-like rate constant the denominator would consist of a 1 added to an exponent (i.e., *a*/(1 + *b* · exp(*v*/*c*)), thus eliminating additional redundancies. Finally, in simulated experiments, the temperature variable was set to 22 °C and calculated accordingly, producing the equations:

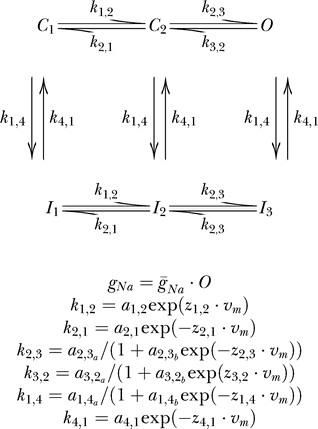
Maximal conductances for simulated experiments were defined as previously described. Again, when fitting experimental data the reversal potential for sodium ions was added as the twentieth variable (Figure 6)

#### Model E.

A 16-parameter Markov model designed for the giant squid axon sodium conductance [16]. As before, redundancies were eliminated. However, we used Patlak's calculations and assumptions to reduce the number of exponential variables to three. Further, while the rate constants and exponential variables were defined as previously described, the nomenclature for the pre-exponential variables was that used by Patlak, producing the equations:

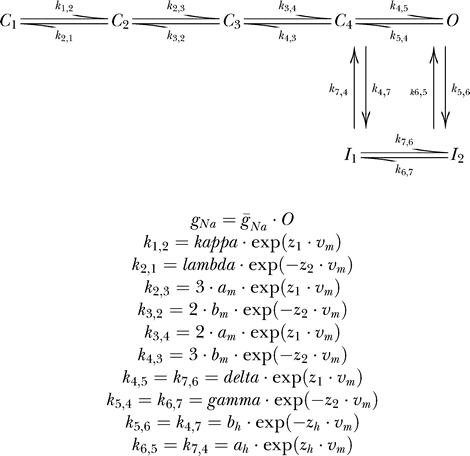



As in preceding models, four maximal conductances were added to the model for each stimulation protocol. Finally, when fitting experimental data the reversal potential for sodium ions was added as the sixteenth variable (Figure 6).

#### Model F.

A 15-parameter Hodgkin-Huxley–like model was based on disjoint analysis of voltage-gated Na^+^ currents recorded from nucleated patches in L5 pyramidal neurons and used for simulating back-propagating action potentials in bitufted interneurons [26].

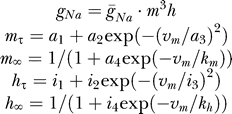
As in preceding models, four maximal conductances were added to the model for each stimulation protocol. Finally, when fitting experimental data the reversal potential for sodium ions was added as the sixteenth variable.

### Slice Preparation

Slices (sagittal, 300 μm thick) were prepared from the somatosensory cortex of 13–15 days old Wistar rats that were killed by rapid decapitation, according to the guidelines of the Bar-Ilan University animal welfare committee. Slice preparation followed Stuart [27]. Slices were perfused throughout the experiment with an oxygenated artificial cerebrospinal fluid (ACSF) containing: (mM) 125 NaCl, 25 NaHCO_3_, 2.5 KCl, 1.25 NaH_2_PO_4_, 1 MgCl_2_, 2 CaCl_2_, and 25 Glucose (pH 7.4 with 5% CO_2_). All experiments were carried out at room temperature (20–22 °C). Pyramidal neurones from L5 in the somatosensory cortex were visually identified using infrared differential interference contrast (IR-DIC) videomicroscopy [27].

To record voltage-gated K^+^ currents, the standard pipette solution contained (mM): 125 K-gluconate, 20 KCl, 10 HEPES, 4 MgATP, 10 Na-phosphocreatine, 0.5 EGTA, and 0.3 GTP (pH 7.2 with KOH). 10 mM 4-AP was included in the bath solution to reduce the amplitude of the A-type K^+^ conductance [28]. The pipette solution for recording voltage-gated Na^+^ currents contained (mM) 120 Cs-gluconate, 20 CsCl, 10 HEPES, 4 MgATP, 10 Na-phosphocreatine, 1 EGTA, and 0.3 GTP (pH 7.2 with CsOH). In addition, 30 mM TEA was added to the bath solution to reduce residual K^+^ current amplitude. A similar amount of NaCl was removed from the bath solution to maintain constant osmolarity.

### Nucleated Outside-Out Patches

Nucleated outside-out patches [29] were extracted from the soma of L5 pyramidal neurons. Briefly, negative pressure (180–230 mbar) was applied when recording in the whole-cell configuration, and the pipette was slowly retracted. Gentle and continuous retraction created large patches of membrane engulfing the nucleus of the neuron. Following extraction of the patch, the pressure was reduced to 20–40 mBar for the duration of the experiment. All measurements from nucleated and cell-attached patches were carried out with the Axopatch-200B amplifier (Axon Instruments, http://www.axon.com). The capacitive compensation circuit of the amplifier reduced capacitive transients. Nucleated patches were held at −60 mV unless otherwise stated. Linear leak and capacitive currents were subtracted off-line by scaling of 20–30 average pulses measured during hyperpolarization (−80 to −100 mV). Currents were filtered at 5–10 kHz and sampled at rates two to ten times higher than the filtering frequency. The reference electrode was an Ag-AgCl pellet placed in the pipette solution and connected to the experimental chamber via an agar bridge containing 150 mM KCl. Under these conditions, the total voltage offset due to electrode and liquid junction potentials [30] was 2 mV. Membrane potential was not corrected for this potential difference. Recordings were made with fire-polished Sylgard-coated (General Electric, RTV615) pipettes (5–8 MΩ).

## Results

One of the main issues concerning GAs (or any minimizing algorithm) is computing power and, consequently, execution times. One of the main goals, when dealing with minimization algorithms, is therefore to produce algorithms which are non-cumbersome, while retaining their ability to efficiently sample the parameter space. A common method for achieving this goal is to limit the parameter search space, for example by adaptive trimming down of the ranges for parameter search [31]. These methods did not reduce the complexity and time detriments of our algorithm.

Instead, while running and analyzing the performance of the GA, we observed a recurring behavior. In 14 preliminary runs, the GA demonstrated a comparable pattern of convergence, where after several hundred generations the best individual in each generation quickly converged to a region in the parameter space. This is illustrated in Figure 1, which describes the steps taken by each parameter of the best individual in each generation in a nine-parameter model. The steps toward each target value are displayed as the percentage change from that parameter value in the previous generation. Figure 1A displays the steps taken by the parameters over the first two hundred generations of the GA progress. During the first fifty generations, large changes were observed in the values of some of the parameters in the best individual vector.

In the further generations, parameter values in the best individual changed only in small steps. Figure 1B shows a detailed view of the algorithm convergence presented above, showing clearly that after several hundred generations the parameters have mutated to only a few percent from their values in the previous generation. Since the parameter search space remained constant, the small changes in value as parameters converged became more insignificant in comparison with the full range of the parameter search space.

We solved this problem by defining a new range for each parameter mutation search space using a Gaussian centered on the best value of that parameter obtained in the previous generation with a relative variance of 5%. The randomly picked value was then multiplied by the current value of each individual found by the GA, providing a new value limited to ∼±22% of the current value of each individual (Figure 2A). The Gaussian range was implemented only after the GA went through at least 500 generations, taking advantage of the fast convergence observed in the initial stages of the run. This new adaptive range for parameter mutation search space proved more efficient than the previously attempted fixed ranges. It increases the probability of the algorithm searching for better individuals around the best value of the individual found so far, as was indeed observed in the preliminary runs. At the same time the possibility of a better individual existing farther away is not dismissed (as the Gaussian is infinite on both ends). A comparison between the GA with and without the adaptive Gaussian range revealed that, while the original algorithm gradually converged and reached a score of 0.1 after 10,000 generations (Figure 2B, smooth line) for the nine-parameter Model A described in the Methods, the new GA converged after 1,000 generations to a score of 10^−4^ (Figure 2B, dashed line).

Subsequently, we simulated potassium currents using several basic kinetic models for potassium channels (see the Channel Models section in Methods). The potassium current data were saved and used as a reference for the GA convergence. We initially tested a nine-parameter model (Model A) with equal value parameters. This did not prevent the resulting simulated currents from resembling experimentally recorded ones. Figure 3A displays the activation of the conductance in response to 20 mV voltages steps from −80 to +60 mV. Figure 3B shows the deactivation of the conductance after 20 ms activation to +60 mV from potentials ranging from −120 mV to −20 mV in steps of 10 mV. This entire set of data was used as the target dataset for calculating the score function (Equation 1) for each individual generated by the GA. After approximately 5,000 generations, the GA converged all of the nine parameters to within 40% of their true target (Figure 3C).

After the GA run was stopped, the data were run through a hill-climbing Principle Axis algorithm (PrAxis) [32], which is part of the NEURON simulation environment. This converged the remaining parameters to 1%–2% of the target values (Figure 3C). To determine the efficiency of the PrAxis routine alone, we generated 100 random parameter vectors and used them as starting guesses for this routine. In none of the cases was the PrAxis routine able to provide even a rough fit of the data. Thus, it is only the combination of the GA followed by the PrAxis that produced a good fit.

We further simulated potassium currents using a thirteen-parameter model (Model B). Again, the potassium current data for activation (Figure 4A) and deactivation (Figure 4B) was saved and used as the target dataset for the GA convergence. As in the previous experiment, after ∼5,000 generations the GA converged all but two parameters to within 40% of their target values (the remaining two reaching values less than 200% of their target value). Once more, 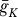 (being independent of membrane potential) converged to within a few hundredths of a percent from its target value. As before, the GA data were run through PrAxis, producing a fit that was only a few percent from the target value for all parameters (Figure 4C). Similar results were obtained for several different target parameters sets.

Using the dataset produced by Models A and B, we also investigated the ability of simulated annealing (SA) and random sampling to replace the GA scheme we present here. The SA algorithm performed as efficiently as the GA in some cases and much worse in others (simulations not shown). Analysis of the simulations revealed that, unlike the GA, the cooling scheme of the SA algorithm had to be repeatedly fine-tuned to properly constrain the parameters of different models. We also tested the ability of extensive random sampling as a substitute for the GA. After 1,000,000 iterations, the best score obtained for Model B was ∼6,000, at least two orders of magnitude bigger than the score obtained by the GA after a similar number of iterations.

**Figure 7:**
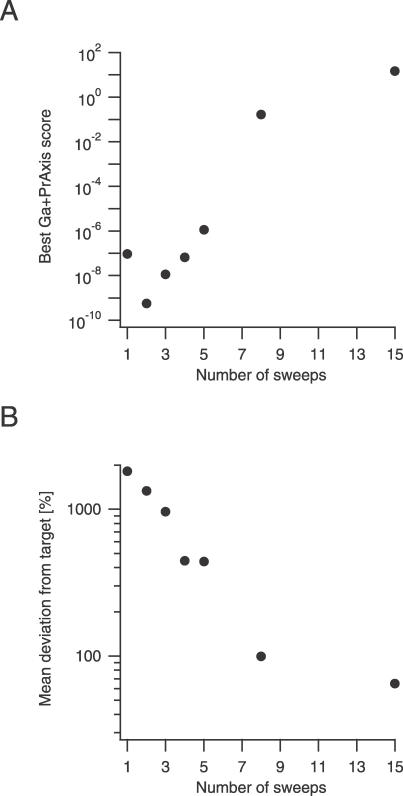
Effect of Increasing Number of Sweeps on GA Performance (A) Best GA and PrAxis fit function score versus an increasing number of activation stimulation sweeps using Model B (as depicted in Figure 4A). (B) Mean deviation of calculated parameter values from their target values versus increasing number of activation stimulation sweeps.

Since our analysis uses a large amount of data, we tested its accuracy in converging on the target parameters using varying amounts of data. We compared the accuracy of the combined convergence of the GA and PrAxis for an increasing number of stimulation sweeps (Figure 9) previously used with Model B to produce the activation traces in Figure 4A. The score function produced a seemingly near-perfect fit when using a small number of stimulation sweeps, but its divergence from zero increased as the number of sweeps grew (Figure 7A). However, an opposing trend was observed when comparing the mean deviation of the best parameter values reached by the GA and PrAxis from their target values (Figure 7B). We therefore conclude that an increasing number of data sweeps is needed for an accurate estimate of the kinetic parameters of a model and does not lead to overfitting of the model. This conclusion was further supported by simulations run on Model C, which proved that an increasing number of stimulation protocols, expressing varying aspects of channel kinetics, also add to the accuracy of the process (unpublished data).

**Figure 8:**
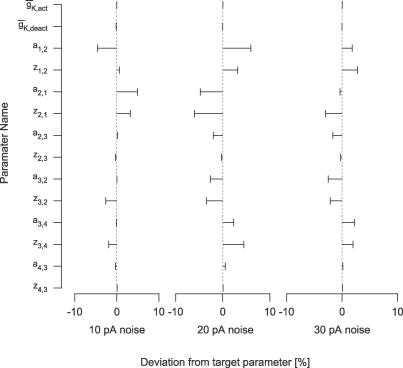
Effect of Noise on GA Performance The errors in the best parameter set obtained by the GA followed by PrAxis plotted relative to their target values (dotted lines) for three cases of increasing white noise added to the simulated currents.

As our aim was to use our GA in fitting a model to currents recorded from neurons, we needed to test our GA's ability to converge in the presence of noise. We therefore created several simulations consisting of randomly generated noise of varying amplitudes. We first tested a 14-parameter model with equal parameters in the presence of random noise whose amplitude was 5% of the current value. (This model was identical to the 13-parameter Model B plus a maximal conduction density for the deactivation protocol, simulating variability between consecutive recordings or, alternatively, data obtained from two different recordings.) The final convergence of both GA and PrAxis resulted in a fit of an average of 5% from the true values (fit not shown). We then ran three similar experiments with constant amplitude noise varying between ±10, ±20, and ±30 pA in each experiment. The results illustrate the GA's ability to converge in spite of the noisy data (Figure 8). The average distance of the parameters from their target values was 1.4%, 2.5%, and 1.4% for the three noise levels respectively.

The response of voltage-gated channels to changes in membrane potential is traditionally measured using a step change in the membrane potential. This is the essence of the voltage-clamp method, which stems from the relative ease of analytically solving the differential equations describing channel relaxation following step activation. Here we have used numerical methods to solve the equations describing channel activation. Therefore, it occurred to us that the GA may also be able to estimate the parameters of a model using a set of data obtained with not-so-standard voltage-clamp protocols. One such protocol is the voltage ramp, which is appealing mainly from the experimental point of view. The voltage ramp is a very useful protocol since it allows the experimentalist a glimpse of the full voltage dependence of a channel using one fast protocol. Furthermore, of all voltage-clamp protocols, the voltage ramp is unique in that during the ramp the contribution of the capacitance to the current is constant. This allows simpler and cleaner leak subtraction than when a square pulse is applied and the capacitive current approaches infinity at the onset of the pulse.

**Figure 9:**
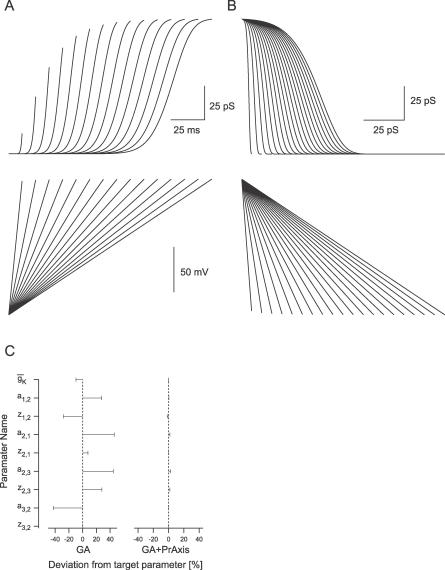
Constraining a Model Using Voltage Ramps (A) The activation (top traces) of a nine-parameter model of a voltage-gated K^+^ channel (Model A) in response to ramps from −100 mV to +50 mV with varying duration (bottom traces). (B) The response of the same model to deactivating voltage ramps from +50 to −100 mV (following a 50-ms step to +50 mV to allow for full-channel activation). (C) The errors in the best parameter set obtained by the GA (left) and GA followed by PrAxis (right) plotted relative to their target values (dotted lines).

Figure 9A displays the activation (top traces) of a nine-parameter model of a voltage-gated K^+^ channel (Model A) in response to ramps of varying duration with potential ranging from −100 mV to +50 mV (Figure 9A, bottom traces). Figure 9B displays the response of the same model to deactivating voltage ramps from +50 mV to −100 mV (following a 50-ms step to +50 mV to fully activate the conductance). The dataset displayed in Figure 9A and 9B was used as target dataset to the GA to determine whether it can be used to constrain the parameters of Model A. After 5,000 generations, the error in the parameters, relative to the target parameters, ranged between 0.01 to 42% (Figure 9C). When this set of parameters was used as an initial guess for the PrAxis hill-climbing algorithm, the error range was reduced to between 10^−4^% to 5% (Figure 9C). Similar results were obtained when Model B was used to generate the target dataset. Thus, this set of simulations demonstrated that the GA could locate the global minimum even when the target dataset was generated using non-classical voltage-clamp protocols.

Following our successful simulations of potassium channel models, we proceeded to more complex simulations of sodium channels. One model tested was based on the sodium current recorded from retinal ganglion cells [24]. The small changes we made to the model (Model C in Methods) were necessary to eliminate redundancy in the original model, which would have prevented the GA from constraining the parameters to the appropriate values. When considering a voltage-gated channel that displays both activation and inactivation, the repertoire of voltage protocols increases substantially. The five basic voltage protocols routinely applied in such cases are activation (Figure 5A), deactivation (Figure 5B), steady-state inactivation (Figure 5C), pulse inactivation (Figure 5D), and recovery from inactivation (Figure 5E). Using this target dataset, the GA, after ∼11,000 generations, generated a parameter set that deviated from the target parameter set by 10%–200% (Figure 5F). Using this set of parameters as an initial guess for the PrAxis hill-climbing algorithm reduced the error range from 10^−4^% to 10^−2^%, practically a perfect fit (Figure 5F).

**Figure 10:**
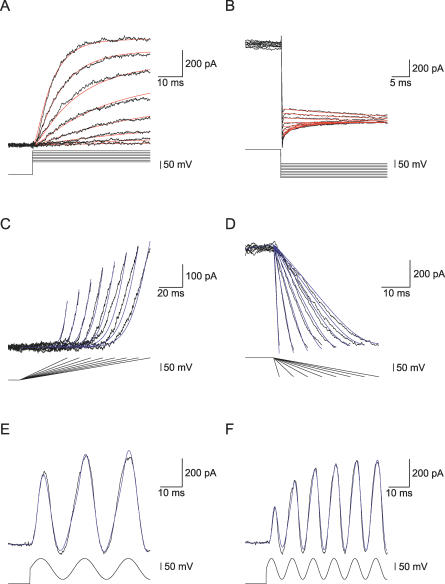
Fitting a Model to Experimentally Recorded Voltage-Gated K^+^ Currents from Neocortical Pyramidal Neurons (A) Outward currents recorded from a nucleated patch. 400-ms pulse to −110 mV followed by a 90-ms pulse to voltages between −80 mV and +80 mV at 20-mV increments. The −110 mV pre-pulse was truncated to facilitate the display of the outward current. Sampled at 20 kHz and filtered at 5 kHz. Leak was subtracted off-line. The red lines are the fit obtained by GA followed by PrAxis for a model with two closed states and one open state (see the text and Table 1). (B) The outward current was generated by a 20-ms voltage step to +80 mV following a 400-ms pre-pulse to −110 mV (truncated). The voltage was then stepped from −120 mV in steps of 10 mV to record the deactivation kinetics. The bath solution contained 10 mM 4-AP and 65 mM K^+^. Leak was subtracted off-line. Filtered at 10 kHz and sampled at 50 kHz. The red lines are the fit obtained by GA followed by PrAxis for a model with two closed states and one open state (see the text and Table 1). The parameters that generated the fit of Model A were: g_K,act_ = 5.95; g_K,deact_ = 4.52; a_1,2_ = 0.07; z_1,2_ = 0.041; a_2,1_ = 0.434; z_2,1_ = 0.021; a_2,3_ = 0.056; z_2,3_ = 0.025; a_3,2_ = 0.011; and z_3,2_ = 0.0317. (C) The outward current was generated by a 500-ms pulse to −100 mV (truncated) followed by voltage ramps to +40 mV. Ramp duration started at 40-ms duration and was increased in steps of 10 ms. The blue lines are the simulated currents produced in response to an identical protocol using Model A with the parameter values detailed in (B). (D) The outward current was generated by deactivating voltage ramps from +40 mV to −80 mV. Ramp duration started at 2 ms and increased in steps of 5 ms (following a 40-ms step to +40 mV to allow for full-channel activation). The blue lines are the simulated currents produced in response to an identical protocol using Model A with the parameter values detailed in (B). Filtered at 5 kHz and sampled at 10 kHz. (E) The outward current was generated using a sinusoidal voltage change following a 600-ms pre-pulse to −100 mV (truncated). The sine wave ranged from +70 mV to −70 mV with a frequency of 50 Hz. The blue lines are the simulated currents produced in response to an identical protocol using Model A with the parameter values detailed in (B). Filtered at 5 kHz and sampled at 10 kHz. (F) The outward current was generated using a sinusoidal voltage change following a 600-ms pre-pulse to −100 mV (truncated). The sine wave ranged between +70 mV to −70 mV with a frequency of 100 Hz. The blue lines are the simulated currents produced in response to an identical protocol using Model A with the parameter values detailed in (B). Filtered at 5 kHz and sampled at 10 kHz.

Next we tested the GA on voltage-gated K^+^ currents recorded in nucleated outside-out patches extracted from layer 5 neocortical pyramidal neurons. These neurons contain several voltage-gated K^+^ channels [28,33]. To reduce the number of channels, we blocked the A-type K^+^ channel with 10 mM 4-AP. The residual current, the slow voltage-gated K^+^ channel [28,33], was activated by voltage steps from −80 to +40 mV (Figure 10A). All the traces in Figure 10 were recorded with an extracellular K^+^ concentration of 6.5 mM to generate larger deactivating tail currents. The activation and deactivation data traces were used as the target dataset for the GA, and the fitness of several models was tested. Following convergence of the GA, the best parameter set was used as an initial guess for the PrAxis routine. The results of these minimizations are summarized in Table 1 where models are compared using their LER [22]. The best fit was obtained for a model with three closed states and one open state in which the rate constants were exponential. However, the fitness of this model, containing 21 free parameters, did not differ substantially from a model with two closed states and only 11 parameters. Thus, to avoid overfitting, we decided that the latter model produced the best fit for this dataset. This fit is shown in Figure 10A and 10B as red lines. Similar results were obtained in two more patches.

Next, we investigated whether the model obtained in this way could predict the response of the conductance to other, less traditional, voltage-clamp protocols. Responses to activation ramps from −100 mV to +40 mV, with varying durations starting from 40 ms and increasing in steps of 10 ms (blue lines), were simulated. These were compared with currents recorded from the patch used in Figure 10A and 10B using identical voltage-clamp protocols (Figure 10C). Simulations of responses to deactivation ramps from +40 mV to −80 mV with durations increasing in 5-ms steps from 2 ms (blue lines) were similarly compared with recorded current traces (Figure 10D). Figure 10E and 10F depict a further comparison of data recorded from the patch and data simulated on the best model using the stimulation protocols shown in Figure 10A and 10B. The voltage-clamp protocols in Figure 10E and 10F were sine waves from −70 mV to +70 mV with a frequency of 50 Hz and 100 Hz, respectively. Again, the blue lines depict the current produced by Model A which had the best fit to the activation and deactivation data in Figure 10A and 10B.

Subsequently, we used the GA on voltage-gated Na^+^ currents recorded in nucleated outside-out patches extracted from layer 5 neocortical pyramidal neurons. Data traces from activation, steady-state inactivation, pulse inactivation, and recovery from inactivation were used as the target dataset for the GA, and the fitness of several models was tested. The activation, steady-state inactivation, and pulse inactivation currents of the voltage-gated Na^+^ channel are shown in Figure 6. The activation currents were produced in response to voltage steps from −30 to +35 mV in steps of 5 mV from a holding potential of −110 mV. The steady-state inactivation currents were produced in response to a voltage step to 0 from holding potentials varying from −105 mV to −5 mV in 10-mV increments. The pulse inactivation currents were produced in response to a voltage step to −50 mV (from a holding potential of −110 mV) for durations varying from 0 ms to 45 ms in increments of 5 ms followed by another voltage step to 0 ms for 30 ms. After convergence of the GA, the best parameter set was used as an initial guess for the PrAxis routine. The results of these minimizations are summarized in Table 2 where models are compared using their LER. The best fit was obtained for a model containing two closed states, an open state, and two inactivated states (Model D, see Figure 6). Examination of the visual fit reveals that Model D has a more accurate description of the channel inactivation, while Model E (Figure 6) is slightly better in depicting the channel activation.

**Table 2:**
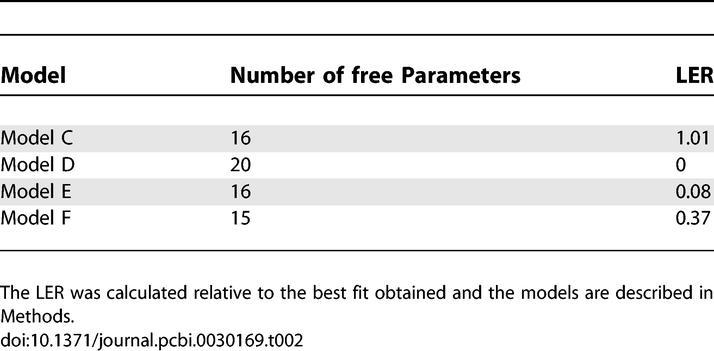
Models Tested in the Fit of the Na^+^ Currents Displayed in Figure 6

## Discussion

Our study describes a method for analyzing voltage-dependent ionic currents. We tested and affirmed the ability of the GA presented here to fit current traces using the full-trace analysis method. The GA was then used to test the fit of various previously published and new voltage-dependent ion channel models (Figures 3,4,7–9). The models were further used to produce currents equivalent to the potassium (Figure 10) and sodium currents (Figure 6) measured in patch-clamp experiments. We conclude that it is possible to verify the viability of voltage-dependent ion channel models using a genetic optimization algorithm concurrently with a full-trace fit of experimental data to the model.

The advantage of our scheme over the more commonly used gradient descent algorithms is that GAs do not require an initial guess of the parameters [12,34,35]. With a large enough dataset, it is possible to arrive at the global minimum even from a random starting point. This is very important since, given wrong starting parameters, gradient descent algorithms may arrive at a local minimum. However, although this study obtained good results in almost all cases, this does not constitute a proof that this method will work each time, since there may be a random combination of parameters that defy the GA. Still, our results show that a useable and functionally significant ion channel model describing whole-cell currents may be produced using a GA with a dataset containing a “complete” whole-cell activation of the channel as its input.

In contrast to the *disjoint* conventional method, the approach to data analysis and model fitting used here has been designated global curve fitting or the *full-trace* method [12,34]. However, the analysis presented here shows several important differences from previously suggested full-trace analyses. The most obvious difference is the use of multiple stimulation protocols; previous full-trace analyses used only activation and deactivation protocols for their data and fit [12,34,35]. Furthermore, while using the full current trace, previous analyses still made the same basic assumptions as their predecessors using the disjoint method, namely the number of gates in a model, the values of m_0_ and h_∞_ in relation to time constants and peak conductance (when dealing with Hodgkin-Huxley–like channel models). Additionally, either due to experimental constraints or ease of use, the same description for rate functions employed by the disjoint method has been used in previous full-trace analysis experiments. Also, independent time constants were used for each potential step in full-trace fits, much as they are commonly used by the disjoint method.

The analysis presented here made no assumptions similar to those used by the disjoint method, thus negating any possible predispositions toward certain time constant–voltage dependencies, stimulation protocols, or other rate-function–limiting conditions. In addition, it is not necessary to consider each voltage step on its own when calculating the time constant dependence on voltage. As all the voltage steps are simulated and fit at once, the resulting time constants should and do fit the target data at all voltage steps. Also, not only does the full-trace analysis presented here take advantage of the full range of stimulation protocols used for voltage-dependent ion channels (Figure 5), but diverse methods of stimulation can also be considered (Figure 9).

The more complex the channel model (i.e., the more parameters it contained), the longer it took and the more difficult it was for the GA to fit the data to the model within a similar time frame. Markov models took longer for the algorithm to locate and converge on the global minimum area in the parameter space. It is also worth noticing that though our experiments show decent fits within reasonable time frames for models with up to 21 parameters, it would probably be prudent not to use such methods for models consisting of more than fifteen parameters when limited by time and computational overhead (especially considering that neither the maximal conductances nor the reversal potential are voltage-dependent and that the search for their global minimum in the parameter space was much quicker than for the corresponding area of the voltage-dependent parameters). Moreover, it is probably best not to try and fit Markov models with many sequential closed states. The kinetic time constants of closed states that do not contribute to the actual whole-cell current will probably be hard to estimate. To investigate such complex models, it is clear that single-channel analysis is more suitable than the approach we have used [13].

Even though we present here a relatively good fit of several models, these models are phenomenological and may not fully describe the gating of the channels. A much more detailed investigation is required to produce a more accurate model of any channel. Such investigation will most probably entail both whole-cell and single-channel recording and analysis. Moreover, sequencing of the channel cDNA and introducing deletions or mutations to the sequence may be required in order to fully determine the structure–function relationship. However, our approach may be used for rapid evaluation of models, which may speed the production of physiologically relevant models.

One forte of the presented approach is most visible when comparing the fit of two models to the same dataset (Figure 6). Both Model D and Model E fit the activation of the channels reasonably well. However, Model D also fits the steady-state inactivation and pulse-inactivation data, whereas Model E fails to fit this portion of the data. Had we used only a dataset containing the activation in the fit, these differences between the two models would not have been exposed. This emphasizes that no fitting routine can replace a rigorous investigation of channel kinetics. Such investigation is crucially required to determine the extent of the dataset required to faithfully represent all the kinetic behavior of the channel. For example, using only activation and deactivation protocols would do injustice to complex processes such as C-type inactivation of voltage-gated K^+^ channels [36,37], the slow inactivation of T-type Ca^2+^ channels [38], the dependence of channel gating on the concentration of ions in the solution [39,40], and many other unique kinetic properties.

Our approach also allows the use of nonstandard voltage-clamp protocols. As recently noted [15], some parameters that may appear in models of voltage-gated channels may be better constrained when the fit is carried out on currents recorded using action-potential trains as a voltage-clamp command instead of the standard voltage-clamp protocol. Admittedly, the standard voltage-clamp protocols facilitate intuitive analysis of the data. Thus, a combination of standard voltage-clamp protocols, used for initial identification of the investigated conductance, with less-standard protocols, used for constraining a model for that conductance, may prove a beneficial approach for future investigations of voltage-gated conductances.

Finally, our analysis technique is automatic. Thus, while the GA searches parameter space for the best combination, the investigator is free to perform more experiments or to design additional models. This is an improvement over the previous disjoint method where every trace had to be manually analyzed, which is inefficient and needlessly time-consuming.

## Supporting Information

Text S1 Demonstration Code A functional NEURON code of the GA described in the manuscript.
(569 KB ZIP)

Video S1 Genetic Algorithm Animation Animation of the convergence of the GA to a model of a voltage-gated ion channel.
(1.0 MB AVI)

## Abbreviations

GA: genetic algorithm: LER, Log Error Ratio
PrAxis: Principle Axis algorithm
SA: simulated annealing
